# Supplementary material for: Factors impacting a patient’s selection of an otolaryngologist
Source: PLoS One. 2025 Dec 9;20(12):e0338793. doi: 10.1371/journal.pone.0338793 (PMC12688101; doi:10.1371/journal.pone.0338793)
Supplement: S1 Table — Significant outcomes of ordinal regression analysis are displayed. The regression model included nine predictors: age, gender, race, education, income, insurance, health status, healthcare worker status, and previous ENT. To account for multiple comparisons, a Bonferroni correction was applied, adjusting the significance threshold to p < 0056. Footnotes specify variable name and associated reference category. aAge; < 25 years old. bGender; Male. cRace; White or Caucasian. dEducation; High school diploma or GED. eIncome; < $30,000. fOverall health status; Excellent health status. gHealthcare worker; Not employed in healthcare. Note: Insurance (Reference: Private/commercial) and Previous ENT (Reference: No previous ENT) were not significant predictors of any preference score. (DOCX) [file pone.0338793.s001.docx]

**S1 Table. Significant predictors of patient preference scores identified by ordinal regression analysis at p < .0056.**

| Survey Question | Factor | Increase in Score (95% CI) | p value |
| --- | --- | --- | --- |
| Surgeon’s educational background (institution where they trained) | American Indian or Alaska Native^c^ | 2.60 (1.43, 3.78) | <.001 |
| Years of experience the surgeon has | American Indian or Alaska Native^c^ | 2.13 (1.05, 3.22) | <.001 |
|  | $90,000+^e^ | 2.39 (1.16, 3.61) | <.001 |
|  | $60,000-$90,000^e^ | 1.78 (0.74, 2.82) | .001 |
|  | $30,000-$60,000^e^ | 1.75 (0.75, 2.75) | .001 |
| Length of time until surgeon has appointment availability | 65+ years old^a^ | 4.30 (2.34, 6.27) | <.001 |
|  | American Indian or Alaska Native^c^ | 3.15 (2.04, 4.27) | <.001 |
|  | $90,000+^e^ | 1.93 (0.76, 3.11) | .001 |
|  | $30,000-$60,000^e^ | 1.66 (0.68, 2.65) | .001 |
| Friends/family recommending the surgeon | 65+ years old^a^ | 3.04 (1.22, 4.86) | .001 |
|  | American Indian or Alaska Native^c^ | 2.15 (1.03, 3.28) | <.001 |
| Reputation of surgeon’s hospital or group | 65+ years old^a^ | 3.65 (1.70, 5.59) | <.001 |
|  | American Indian or Alaska Native^c^ | 2.05 (0.92, 3.18) | <.001 |
|  | $30,000-$60,000^e^ | 1.49 (0.50, 2.47) | .003 |
| Marketing (billboards, ads) | $90,000+^e^ | 1.88 (0.65, 3.11) | .003 |
|  | $30,000-$60,000^e^ | 1.55 (0.49, 2.61) | .004 |
|  | Employed in healthcare^g^ | -1.54 (-2.17, -0.91) | <.001 |
| Surgeon states that they use new surgical technologies or techniques | 65+ years old^a^ | 2.96 (1.20, 4.72) | .001 |
|  | American Indian or Alaska Native^c^ | 2.43 (1.28, 3.59) | <.001 |
|  | Good health status^f^ | -1.05 (-1.72, -0.37) | .002 |
| Surgeon’s or practice’s website | Female^b^ | -0.72 (-1.18, -0.27) | .002 |
|  | American Indian or Alaska Native^c^ | 2.47 (1.38, 3.57) | <.001 |
|  | $30,000-$60,000^e^ | 1.71 (0.72, 2.71) | .001 |
|  | Employed in healthcare^g^ | -1.04 (-1.63, -0.44) | .001 |
| Surgeon’s Twitter posts | American Indian or Alaska Native^c^ | 1.74 (0.65, 2.83) | .002 |
|  | Good health status^f^ | -1.20 (-1.88, -0.52) | .001 |
|  | Employed in healthcare^g^ | -1.27 (-1.92, -0.62) | <.001 |
| Surgeon’s Facebook posts | American Indian or Alaska Native^c^ | 1.88 (0.79, 2.96) | .001 |
|  | $30,000-$60,000^e^ | 1.37 (0.40, 2.34) | .005 |
|  | Good health status^f^ | -1.27 (-1.94, -0.61) | <.001 |
|  | Employed in healthcare^g^ | -1.00 (-1.66, -0.35) | .003 |
| Surgeon’s Instagram posts | American Indian or Alaska Native^c^ | 2.02 (0.89, 3.16) | <.001 |
|  | $30,000-$60,000^e^ | 1.66 (0.61, 2.72) | .002 |
|  | Good health status^f^ | -0.99 (-1.65, -0.33) | .003 |
|  | Employed in healthcare^g^ | -1.13 (-1.77, -0.50) | <.001 |
| Favorable office location | Black or African American^c^ | 1.58 (0.56, 2.59) | .002 |
| Ease of parking | Good health status^f^ | -0.93 (-1.57, -0.29) | .005 |
| Surgeon’s online reviews (Healthgrades, Google reviews, Yelp, etc.) | Good health status^f^ | -0.94 (-1.59, -0.28) | .005 |
| Professionalism of office staff | 65+ years old^a^ | 3.85 (1.88, 5.81) | <.001 |
|  | Female^b^ | -0.68 (-1.14, -0.23) | .003 |
|  | American Indian or Alaska Native^c^ | 2.39 (1.24, 3.53) | <.001 |
| Quality of the surgeon’s office/hospital facilities and buildings | American Indian or Alaska Native^c^ | 1.94 (0.86, 3.03) | <.001 |
|  | Associates or technical degree^d^ | -3.10 (-4.84, -1.36) | <.001 |
|  | $90,000+^e^ | 1.88 (0.65, 3.12) | .003 |
| Surgeon’s attire/clothing | American Indian or Alaska Native^c^ | 2.71 (1.56, 3.85) | <.001 |
| Surgeon’s gender | Good health status^f^ | -1.28 (-1.93, -0.63) | <.001 |
|  | Employed in healthcare^g^ | -1.63 (-2.28, -0.98) | <.001 |
| Surgeon’s professionalism | American Indian or Alaska Native^c^ | 1.79 (0.68, 2.90) | .002 |
| How much time the surgeon spends with you | American Indian or Alaska Native^c^ | 2.57 (1.49, 3.64) | <.001 |
|  | Black or African American^c^ | 1.63 (0.58, 2.69) | .002 |
|  | $30,000-$60,000^e^ | 1.66 (0.70, 2.61) | .001 |
| Ability of the surgeon to grasp your problem | 65+ years old^a^ | 5.51 (2.91, 8.12) | <.001 |
|  | American Indian or Alaska Native^c^ | 2.68 (1.56, 3.80) | <.001 |
|  | Black or African American^c^ | 2.52 (1.41, 3.64) | <.001 |
| Surgeon’s physical examination of you | 65+ years old^a^ | 3.35 (1.53, 5.17) | <.001 |
|  | American Indian or Alaska Native^c^ | 1.62 (0.60, 2.65) | .002 |
|  | Black or African American^c^ | 1.68 (0.60, 2.76) | .002 |
|  | $30,000-$60,000^e^ | 1.63 (0.68, 2.58) | .001 |
| Your ability to see the exam performed by the surgeon (screen with a view of scope/ear exam) | Female^b^ | -0.68 (-1.14, -0.21) | .004 |
|  | American Indian or Alaska Native^c^ | 2.20 (1.15, 3.26) | <.001 |
|  | Black or African American^c^ | 1.85 (0.77, 2.92) | .001 |
|  | $60,000-$90,000^e^ | 1.73 (0.65, 2.81) | .002 |
|  | $30,000-$60,000^e^ | 1.76 (0.72, 2.80) | .001 |
| Surgeon’s explanation of your diagnosis and treatment options | American Indian or Alaska Native^c^ | 2.32 (1.29, 3.36) | <.001 |
|  | Black or African American^c^ | 1.82 (0.73, 2.91) | .001 |
|  | $60,000-$90,000^e^ | 1.35 (0.30, 2.40) | .005 |

Significant outcomes of ordinal regression analysis are displayed. The regression model included nine predictors: age, gender, race, education, income, insurance, health status, healthcare worker status, and previous ENT. To account for multiple comparisons, a Bonferroni correction was applied, adjusting the significance threshold to p < .0056. Footnotes specify variable name and associated reference category.

^a^Age; <25 years old

^b^Gender; Male

^c^Race; White or Caucasian

^d^Education; High school diploma or GED

^e^Income; <$30,000

^f^Overall health status; Excellent health status

^g^Healthcare worker; Not employed in healthcare

Note: Insurance (Reference: Private/commercial) and Previous ENT (Reference: No previous ENT) were not significant predictors of any preference score.
